# Supplementary material for: Polyketide synthase gene clusters in the ericoid mycorrhizal fungus Oidiodendron maius: genome-wide prediction, expression profiling, and functional characterization of knockout mutants
Source: Front Plant Sci. 2026 Sep 16;17:1924430. doi: 10.3389/fpls.2026.1924430 (PMC13623903; doi:10.3389/fpls.2026.1924430)
Supplement: Supplementary file 1 [file SupplementaryFile1.zip › Supplementary Materials/Supplementary Folder 2.zip!/Folder S2/Oidma1_SM_Step10_scaffold_1_MultiOmics_Karyoplot.pdf]

scaffold_1

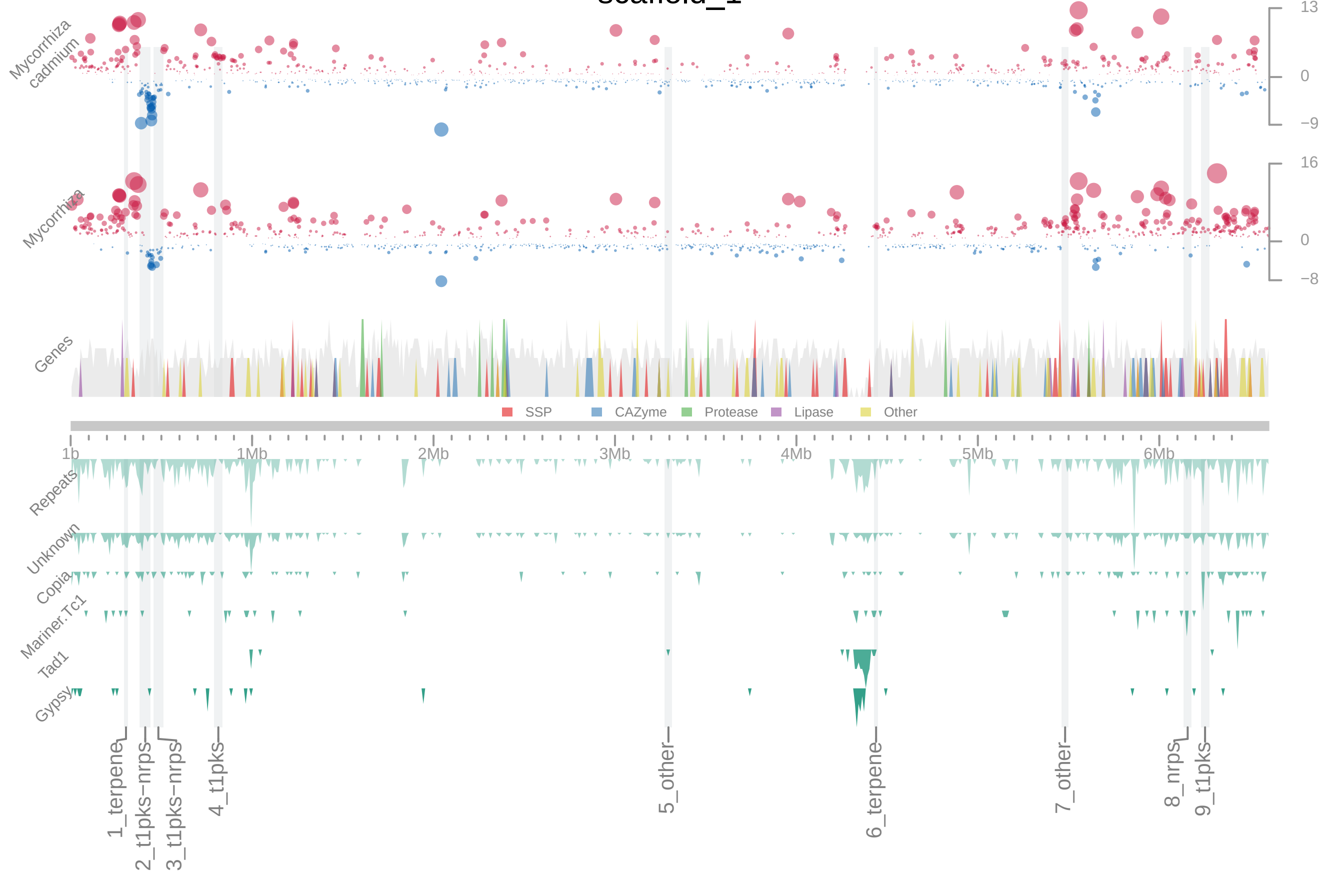
Mycorrhiza
cadmium
Mycorrhiza
Genes
13
0
−9
16
0
−8
SSP
CAZyme
Protease
Lipase
Other
1b
1Mb
2Mb
3Mb
4Mb
5Mb
6Mb
Repeats
Unknown
Copia
Mariner.Tc1
Tad1
Gypsy
1_terpene
2_t1pks−nrps
3_t1pks−nrps
4_t1pks
5_other
6_terpene
7_other
8_nrps
9_t1pks
